# The Role and Impact of Minimal Residual Disease in NSCLC

**DOI:** 10.1007/s11912-021-01131-w

**Published:** 2021-11-04

**Authors:** Daniele Frisone, Alex Friedlaender, Alfredo Addeo

**Affiliations:** 1 Department of Oncology, Geneva University Hospital, Geneva, Switzerland; 2 Clinique Générale Beaulieu, Geneva, Switzerland

**Keywords:** Minimal residual disease, NSCLC, Lung cancer, ctDNA, Adjuvant therapy

## Abstract

**Purpose of Review:**

There has been a huge development in the assessment of malignancies through liquid biopsies last years, especially for NSCLC, where its use has become part of clinical practice in some settings. We aim to summarize current evidence about minimal residual disease and its use in lung cancer.

**Recent Findings:**

Recent studies using ctDNA in NSCLC but also in other types of cancer found strong correlations between the presence of ctDNA and the risk of disease progression or death after curative intent, despite current technical difficulties in performing this analysis (high sensitivity and specificity required).

**Summary:**

Evaluation of MRD in NSCLC, especially through ctDNA, could be an important point in future trial designs and could permit a more “targeted” adjuvant treatment.

## Introduction

Lung cancer is the leading cause of cancer mortality with more than 2 million cases worldwide and 1,750,000 estimated deaths in 2018 [1]. Almost 90% of lung cancer in the Western World is represented by NSCLC [2], which represents indeed a major health problem.

Adjuvant chemotherapy has become standard of care for stages II–III resected NSCLC and for some stage IB with tumour larger than 4 cm. Still, the advantage of adjuvant chemotherapy is scarce (roughly 5%), and the toxicity of standard cisplatin chemotherapy is not negligible [3].

Today, the ability to analyse plasma for circulating tumour cells and circulating tumour DNA (ctDNA) has the potential to change the definition of a complete response or complete remission in non-small cell lung cancer (NSCLC). In some settings, especially among patients with haematological malignancies, the detection of the remaining tumour cells after therapy is referred to as minimal residual disease (MRD). In lung cancer, the impact of these tests, currently in their infancy in terms of clinical practice, could lead to changes in treatment algorithms, both in the adjuvant and metastatic setting.

Here, we discuss currently available data and potential implications of MRD in NSCLC.

## Definition of MRD

MRD in NSCLC can be defined as micrometastases or minimal residual disease that persists after initial therapy. This MRD represents a potential source of subsequent metastatic relapse at distant sites. MRD detection and monitoring are established and widely used in patients with haematological malignancies but remain challenging in patients with solid tumours, owing to a difficulty in sampling the low concentrations of circulating tumour cells (CTCs) or factors shed from the cancer cells into the bloodstream.

## How to Measure MRD

In recent years, liquid biopsy techniques have been developed, making it possible to analyse blood or other fluids for circulating tumour cells, exosomes, RNA and circulating DNA (ctDNA). Currently, the latter is the most useful and studied technique and appears to have great potential as a tool to identify MRD in NSCLC [4].

MRD was first investigated and utilized for haematological malignancies, where there is often a known single gene mutation driving the disease. This has allowed better treatment and follow-up of patients with these kinds of malignancies, along with the development of sensitive testing techniques, such as reverse transcriptase polymerase chain reaction (RT-PCR) [5].

For solid tumours, a wide variety of mutations has been identified, and these vary according to the tumour origin and histologic subtype, making the use of PCR more complicated. The wide-spread use of next-generation sequencing (NGS) has permitted a broader mutational analysis of ctDNA in plasma [6–9], with a very high sensitivity, making it possible to measure MRD in solid tumours.

If tumour cells shed fragments of DNA sequences, these enter the circulation and can be detected by NGS. These sequences can often be differentiated from non-tumoural cell-free DNA (cfDNA), most often released by normal haematological cells [10•].The distinction stems from somatic mutations, both acquired during carcinogenesis or driving it, often leading to sequences not found in healthy tissues [11]. Modern NGS provides the required extremely sensitive detection and discrimination technique, as the proportion of tumour-derived mutant forms of an allele is estimated to be often less than 1% of the total cfDNA [12].

## The Role of MRD in the Adjuvant Setting

In operable stages I–III NSCLC, there is a significant risk of relapse after a surgery with curative intent. For instance, in stage IB disease, there is a 45% risk of relapse, while this rises to 76% in stage IIIA disease [13]. As previously stated, cisplatin-based adjuvant chemotherapy can reduce the risk of relapse by up to 16% and improve 5-year overall survival by roughly 5%. Today, other than disease stage and patient fitness, there are no criteria to assess which patients will truly benefit from adjuvant therapy. This means that the majority of patients will be exposed to the significant toxicity of chemotherapy without benefit.

This is where MRD could play a crucial role. Detecting CTCs or ctDNA post-operatively could predict the risk of relapse and could perhaps permit a more targeted use of adjuvant chemotherapy. There is already some evidence supporting the potential role of MRD in this context.

One study investigated the dynamics and optimal timing to evaluate ctDNA after surgical resection in 36 NSCLC patients, enrolled prospectively. This study demonstrated that ctDNA has a very short half-life of 35 min and that its evaluation is predictive of RFS and OS when assessed on days 3 and 30 after surgery, but not on day 1 [14]. Another study investigated the role of a technique called CAPP-seq (cancer personalized profiling by deep sequencing, very sensitive up to an allele fraction of 0.02% [6]) to evaluate ctDNA in 40 patients with stages I–III lung cancer treated with radical treatment. This study showed that ctDNA was detected in 20/20 patients who ultimately recurred, with the detection preceding radiographic progression by a median of 5.2 months [15]. A recent study involving 77 patients with early-stage NSCLC undergoing surgery found a strong correlation between both pre-operative and post-operative ctDNA positivity with RFS and OS [16].

CtDNA has also demonstrated a strong prognostic value in oesophageal and colorectal cancers [17, 18]. However, one of the current challenges and limitations is that ctDNA must be assessed in patients before radical treatment, and the sensitivity of today’s methods, although high, does not allow for the detection of ctDNA in all patients [19•].

## The Predictive Value of ctDNA on Response to Treatment

Another study from one of the previously cited research groups aimed to verify the utility of ctDNA to predict outcomes after radical chemo-radiotherapy (CRT) in NSCLC, before and after the use of durvalumab as consolidation therapy [20]. In this study, a cohort of 28 patients was treated with consolidation immune checkpoint inhibitors (ICI). Seventy-nine percent of these patients were treated with durvalumab as a standard of care and the remaining 21% with atezolizumab in the context of a clinical trial. Only 23 of 28 patients could be evaluated, since no pretreatment ctDNA was detected in the remaining five patients. This study confirmed the high prognostic value of ctDNA. In particular, patients with detectable ctDNA after CRT but with a decreasing or undetectable ctDNA after the initiation of ICI showed a PFS of 100% at 1 year [21•]. Similar results in the metastatic setting were found in a study involving 28 NSCLC patients treated with ICIs, with a high concordance between ctDNA drop and radiological response, as well as a strong correlation with PFS and OS (HR 0.17, *p* = 0.007) [22].

Similarly, in a small retrospective study, ctDNA analyses appear to play an important role in predicting the risk of disease relapse and long responders to ICIs. Further research is warranted, as ctDNA may be key to adapting the duration of ICIs treatment in clinical practice [23].

## Limitations of MRD (ctDNA)

Pre-analytical considerations are standardized, including the necessity of an EDTA tube and correct measures for plasma extraction and storage. Probably, the most important limitation of ctDNA is analytical in nature, namely the difficulty to detect somatic mutations at an allele frequency permitting the analysis [19•]. For example, in one of the previously cited studies, only 28 out of 49 patients had detectable somatic mutations and could be followed with ctDNA [22].

While PCR-based approaches have a sensitivity reaching 0.01–0.001% of an allele fraction, they are limited by the analysis of a single known point mutation. NGS approaches have a sensitivity of 0.1–1%, while a personalized approach such as CAPP-seq (cancer personalized profiling by deep sequencing) is useful for ctDNA monitoring with a sensitivity as high as 0.00025% of allele fraction, but it is limited by the results of a patient’s tumour mutation analysis [10•, 24].

Finally, especially in the setting of early disease, these issues seem to be more challenging since the release of ctDNA is strongly correlated with the burden of disease [10•]. Moreover, it is important to signal that false positive is possible, due essentially to ageing or other benign conditions and to the fact that the detection of a somatic mutation is not always linked to cancer development [25].

Another issue when speaking about MRD and, in general, of liquid biopsies is the concordance of mutational analyses between plasma analyses and tissue biopsies. While there is often a good concordance, it varies depending on the used method, with ultra-deep NGS appearing to be the best strategy in terms of mutational concordance in advanced tumours [26, 27].

## Future Direction and Trials

The use of ctDNA in lung cancer is rapidly evolving, covering different domains. While the use of liquid biopsies is already a reality for stage IV EGFR mutated cancer, there has been great development in cancer screening or in early disease, where the selection of patients undergoing heavy adjuvant treatments become crucial. [28]

The detection of ctDNA months or years after curative-intent treatment indicates the persistence of MRD. The concentrations of ctDNA in these patients are, however, much lower than in patients with established metastatic disease. Therefore, in early disease, the use of ultrasensitive detection technologies is a necessity [29]. In addition to the choice of the appropriate technology, the volume of blood that is analysed is an important but frequently overlooked pre-analytical variable. CtDNA occurs at very low abundances in patients with early stage cancer, and the analysis of only a 5–10 ml blood sample might not be sufficient to obtain robust results.

**Fig. 1:**
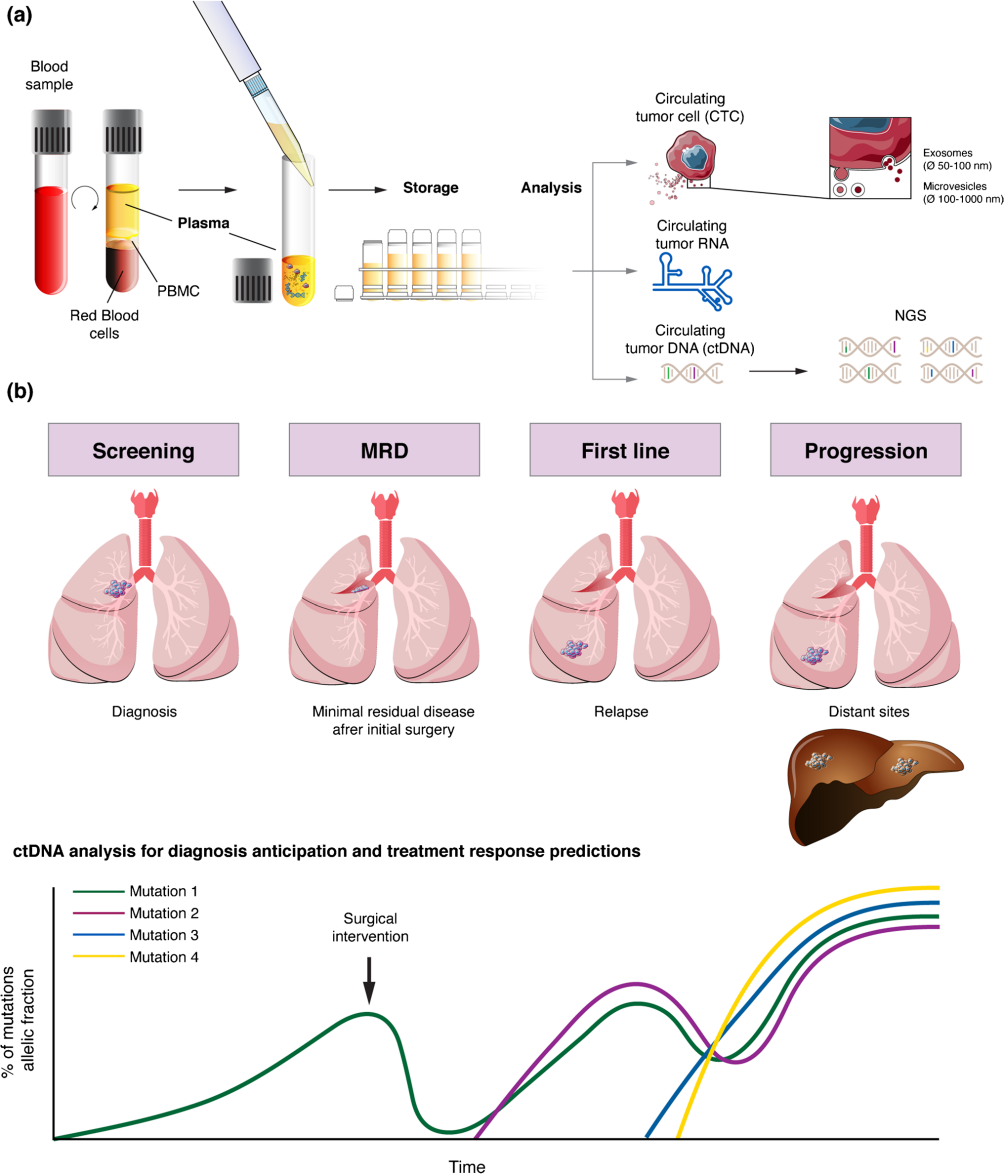
Steps in management of blood samples to liquid biopsies (**a**). General association between disease stage and ctDNA analysis (**b**)

Another important question is whether the assay is sufficient to detect all or only a specific subset of ctDNA code that is captured in the blood sample. For ctDNA assessment in the MRD setting, the genetic analysis of the primary tumour from individual patients enables the development of personalized mutation panels and, thus, the use of technologies with very high analytical specificity and sensitivity (e.g. integrated digital error suppression (iDES)-enhanced CAPP-Seq, which has a ctDNA detection limit of 0.004% in plasma) [30, 31]. By contrast, with early cancer detection platforms, either a broad spectrum of possible mutations must be interrogated, which would decrease the sensitivity and increase costs, or one must acknowledge the risk that tumours with rare mutations will be missed by the assay [28] (Fig. 1).

For CTC analyses in the MRD setting, the current epithelial markers used in most assays to identify carcinoma cells at the single-cell level might fail to detect CTCs that have undergone epithelial-to-mesenchymal transition (EMT) [32]. EMT has important roles in cancer cell dissemination and homing to distant sites, while the reverse process—mesenchymal-to-epithelial transition—is required for outgrowth into overt metastases. Conceivably, therefore, CTCs in patients with MRD following treatment of early stage cancer might have a higher frequency of downregulation of epithelial markers and upregulation of mesenchymal markers compared to CTCs from patients with overt metastases. However, most assays, including the FDA-approved CellSearch system, are able to detect tumour cells with some degree of EMT, perhaps including cancers cells with an intermediate EMT phenotype that are known to have the highest levels of plasticity and stemness [33].

Tumour heterogeneity poses a major challenge to MRD detection. At the present time, the optimal number of different mutations in ctDNA (or in CTCs) that need to be assessed in order to be sure of avoiding false-negative findings remains unclear. Clonal mutations present in the resected primary tumour specimen of each individual patient are obvious candidates for liquid biopsy-based disease monitoring, but subclonal mutations can become drivers of MRD if they confer a natural and/or therapy-induced selective advantage for the survival of tumour cells. False-positive findings are also a concern if the concentration of ctDNA is very low and ultrasensitive methods are applied (e.g. CAPP-Seq). This is because tissue ageing (reflected by clonal haematopoiesis of indeterminate potential) and benign lesions (such as naevi) can result in a low background level of clinically nonsignificant mutations being detectable in the blood [34–36].

Additional technical difficulties facing the field of liquid biopsies include the variability in pre-analytical and analytical conditions that can hinder the application of CTC or ctDNA platforms used for the detection and characterization of MRD in clinical practice. Thus, more emphasis on technical validation is required [37]. Several consortia have been working on this challenge, combining the expertise of academic and industry partners, with the hope of developing robust liquid biopsy assays and designing the trials needed to prove the clinical utility of liquid biopsy testing.

## Conclusion

Assessing MRD is gaining ground and interest in NSCLC, with the development of new, ultra-sensitive types of testing that could change clinical practice in the coming years. Today, MRD will increasingly be a part of trial design. For instance, in the phase III MERMAID-1 trial, MRD-positive completely resected stages II–III NSCLC patients are randomized to adjuvant chemotherapy ± durvalumab, a checkpoint inhibitor (NCT04385368). Another trial evaluating an intensified strategy for adjuvant treatment (with chemo-immunotherapy with nivolumab) only in patients with post-operatively positive ctDNA has been presented recently [38•]. On the other hand, we believe an even greater potential impact of MRD assessment could be evaluated in a prospective trial randomly assigning lung cancer patients with negative post-operative ctDNA assessment to adjuvant chemotherapy versus placebo. Depending on the outcome, this could change practice and help spare toxic chemotherapy for those who may not benefit from it.
